# Attenuation of constitutive DNA damage signaling by 1,25-dihydroxyvitamin D3

**DOI:** 10.18632/aging.100450

**Published:** 2012-04-11

**Authors:** H. Dorota Halicka, Hong Zhao, Jiangwei Li, Frank Traganos, George P. Studzinski, Zbigniew Darzynkiewicz

**Affiliations:** ^1^ Brander Cancer Research Institute and Department of Pathology, New York Medical College, Valhalla, NY 10595; ^2^ Department of Pathology and Laboratory Medicine, New Jersey Medical School, UMDNJ, Newark, NJ 07101

**Keywords:** Calcitriol, histone H2AX phosphorylation, ATM activation, cell cycle, apoptosis, oxidative DNA damage, DNA repair, aging, cancer prevention, replication stress, mTOR

## Abstract

In addition to its traditional role in the regulation of calcium homeostasis and bone metabolism, vitamin D also exhibits immunomodulatory, anti-proliferative and cancer preventive activities. Molecular mechanisms that confer the chemo-preventive properties to vitamin D are poorly understood. We previously reported that constitutive phosphorylation of histone H2AX on Ser139 (γH2AX) and activation of ATM (Ser1981 phosphorylation), seen in untreated normal or tumor cells predominantly in S phase of the cell cycle, is to a large extent indicative of DNA replication stress occurring as a result of persistent DNA damage caused by endogenous oxidants, by-products of oxidative metabolism. In the present study we observed that exposure of mitogenically stimulated human lymphocytes, pulmonary carcinoma A549 and lymphoblastoid TK6 cells to 1,25-dihydroxyvitamin D3 (1,25-VD) reduced the level of constitutive expression of γH2AX and ATM-S1981^P^. We also observed that the H_2_O_2_-induced rise in the level of γH2AX in lymphocytes was attenuated by 1,25-VD. Whereas in lymphocytes 1,25-VD reduced by 50-70% the level of endogenous oxidants as determined by their ability to oxidize 2,7-dichlorodihydrofluorescein (DCFH) in A549 and TK6 cells the attenuation of DNA damage signaling by 1,25-VD was seen in the absence of detectable reduction in DCFH oxidation. These findings suggest that while the anti-oxidant activity of 1,25-VD may contribute to a reduction in the intensity of DNA replication stress in lymphocytes, other factors play a role in the 1,25-VD effects seen in A549 and TK6 cells. The data are consistent with the recent report on the interaction between DNA damage signaling (ATM activation) and 1,25D receptor (VDR) phosphorylation that lead to enhancement of DNA repair efficiency, and provide further support for the chemo-preventive and anti-aging properties of this vitamin/hormone.

## INTRODUCTION

Regulation of calcium homeostasis and bone metabolism is the most widely recognized function of 1,25-dihydroxyvitamin D3 also known as calcitriol (1,25-VD), the hormonal form of vitamin D. However, there is a growing body of evidence showing that 1,25-VD also plays a significant role in the prevention of different types of cancer [1-9]. The molecular mechanisms that may be involved in the chemo-preventive and antitumor activities of 1,25-VD have been extensively reviewed in recent publications [3,9,10]. One of the mechanisms that may contribute to the chemo-preventive properties of 1,25-VD is modulation of the DNA damage response that facilitates successful DNA repair after treatment with genotoxic agents [10-16]. This mechanism was observed particularly after exposure of cells to UV light [11-14]. Recent studies on gene profiling revealed that the DNA repair genes are among a multitude of genes whose transcription is induced by 1,25-VD [17-19].

Another mechanism that may contribute to the chemo-preventive properties of 1,25-VD is likely provided by its antioxidant activity. Persistent DNA damage by endogenous oxidants, by-products of aerobic respiration, is considered to be one of the causes of carcinogenesis and aging [20-24]. Accumulated genomic mutations from improperly repaired DNA, particularly at the sites of oncogenes or tumor suppressor genes, may result in malignant transformation. There is ample evidence that 1,25-VD provides protection against oxidative DNA damage. Thus, constitutive DNA damage in colon epithelium, likely induced by endogenous oxidants, was seen to be elevated in vitamin D receptor (VDR)-knockout mice [25]. Conversely, the treatment of humans with a daily dose of 800 I.U. of vitamin D was shown to reduce oxidative DNA damage in colon epithelium [26]. Treatment of cells of several cell lines with 1,25-VD led to induction of enzymes involved in protection against oxidative damage. One of them is thioredoxin reductase 1 (TXNRD1), the enzyme which maintains thioredoxin in the reduced state needed to confer a reduced intracellular redox state and thereby limit intracellular oxidant stress [27-30]. Another antioxidant protein induced by 1,25-VD is superoxide dismutase (SOD) [28-31].

We have recently reported that untreated normal or tumor cells in culture exhibit a “background” level of DNA damage signaling (DDS) revealed as histone H2AX phosphorylation on Ser139 and activation of *Ataxia Telangiectasiamutated* protein kinase (ATM) through phosphorylation on *Ser*1981 [32-34]. These phosphorylation events, which report DNA damage and in particular formation of DNA double-strand breaks (DSBs) [35,36], were detected in individual cells immunocytochemically using phospho-specific Abs and measured by flow and laser scanning cytometry (LSC) [32-34]. Multiparameter cytometric analysis made it possible to correlate the level of this intrinsic, constitutive DDS, with the cell cycle phase and with the cell's propensity to undergo apoptosis. In a series of experiments we demonstrated that, at least to a large degree, the constitutive DDS is in response to oxidative DNA damage induced by endogenous oxidants produced during aerobic respiration [32-34]. Mitogenic stimulation of lymphocytes dramatically increased the level of constitutive DDS concurrent with an increase in the generation of endogenous oxidants and activation of the mTOR pathway [37]. Conversely, prolonged exposure of cells to agents that reduced the level of endogenous oxidants such as hyaluronan [38], or to metformin, the anti-diabetic drug, inducer of AMP-activated protein kinase and mTOR suppressor [39], led to a significant attenuation of constitutive DDS. In light of previous evidence for the antioxidant and chemo-preventive properties of 1,25-VD, in the present study we explored whether this vitamin/hormone has the ability to affect the level of constitutive DDS.

## RESULTS

**Figure 1:**
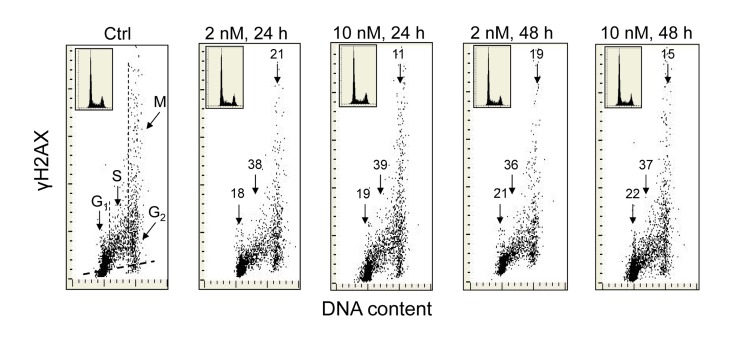
Effect of the treatment of A549 cells with 1,25-VD on the level of expression of γH2AX with respect to the cell cycle phase Exponentially growing human pulmonary carcinoma A549 cells were untreated (Ctrl) or treated with 2 or 10 nM 1,25-VD for 24 or 48 h and expression of γH2AX was detected immunocytochemically using phospho-specific Ab targeting the phosphorylated S139 epitope; the intensity of fluorescence was measured by laser scanning cytometry (LSC) [70]. The numbers above the respective arrows indicate the percent reduction in intensity of immunofluorescence of the mean values of cell populations in G_1_, S and G_2_M phases of the cell cycle (gated as shown in the Ctrl panel) in the 1,25-VD-treated cells with respect to the untreated cells (Ctrl), in the same phase of cell cycle. Inserts show cellular DNA content histograms from the respective cultures. The skewed dash line in Ctrl panel shows the maximal level of fluorescence intensity of the cells stained with isotype control Ab.

Exposure of A549 cells to 1,25-VD at a concentration of 2 or 10 nM for 24 and 48 h led to a distinct reduction in the level of expression of γH2AX (Figure 1). As it is evident from the bivariate DNA content *versus* γH2AX expression dotplots, the degree of decline in γH2AX expression was similar regardless of 1,25-VD concentration and duration of cell treatment. However, a distinct difference was apparent with regard to cell cycle phase. The cells most affected were in S-phase of the cell cycle which showed a reduction in γH2AX within the range of 36% to 39%. The effect of 1,25D was less pronounced in the case of G_1_ or G_2_M phase cells as their γH2AX expression was decreased by only 18-22% or 11-21%, respectively. During the time and at the concentration of 1,25-VD studied here, there was no detectable effect on the cell cycle distribution of A549 cells, as is evident from the similarity of the cellular DNA content histograms shown in the respective insets.

**Figure 2:**
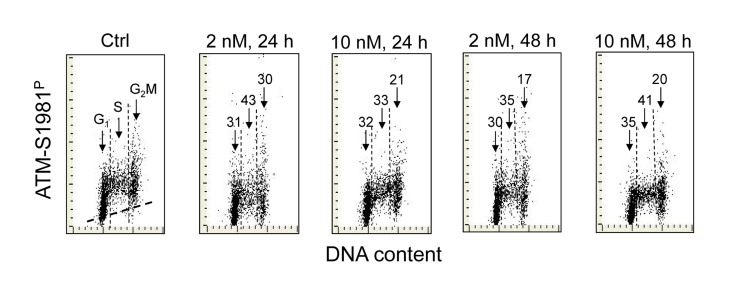
Effect of treatment of A549 cells with 1,25-VD on the level of expression of ATM phosphorylated on Ser1981 in relation to the cell cycle phase Expression of ATM-S1981^P^ in A549 cells treated with 2 nM or 10 nM 1,25-VD for 24 or 48 h was detected immunocytochemically using phospho-specific Abs and measured by LSC. The numbers above the arrows indicate the percent reduction in intensity of immunofluorescence of the mean values of cell populations in G_1_, S and G_2_M phases of the cell cycle in the 1,25-VD-treated cells with respect to the untreated cells (Ctrl) in the same phase of cell cycle. The skewed dash line in Ctrl panel shows the maximal level of fluorescence intensity of the cells stained with isotype control Ab.

Figure 2 illustrates the effect of 1,25-VD on the level of phosphorylation of ATM at S1981 in A549 cells. As in the case of H2AX phosphorylation (Figure 1), the expression of S1981 phosphorylated ATM was markedly reduced in cells exposed to 1,25-VD. The degree of decline in expression of phosphorylated ATM was similar at 2 nM and at 10 nM of vitamin D and following 24 h or 48 h of exposure. As compared with the effect of 1,25-VD on γH2AX expression (Figure 1) there were less pronounced differences in the degree of reduction of ATM-S1981^P^ between the cells in different phases of the cell cycle. However, the cells in S phase were somewhat more affected (33% - 43% reduction) compared to G_1_ (30-35%) or G_2_M cells (17-30%), respectively.

**Figure 3:**
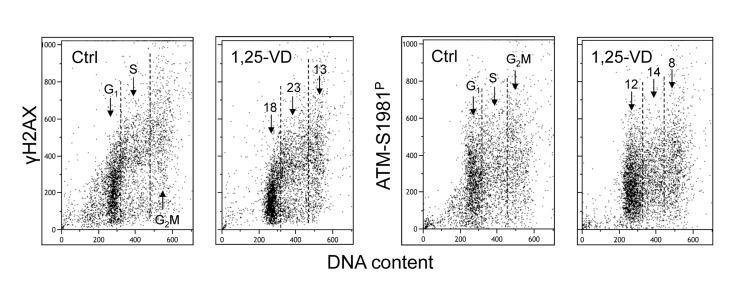
Effect of treatment of TK6 cells with 1,25-VD on the level of constitutive expression of γH2AX and ATM-S1981^P^ Exponentially growing TK6 cells were untreated (Ctrl) or treated with 10 nM 1,25-VD for 24 h. Expression of γH2AX and ATM-S1981^P^ was detected in individual cells immunocytochemically, cellular fluorescence was measured by flow cytometry. The numbers above the arrows show the percent reduction in intensity of immunofluorescence of the mean values of cell populations in G_1_, S and G_2_M phases of the cell cycle in the 1,25-VD-treated cells with respect to the untreated cells (Ctrl) in the same phase of cell cycle.

Treatment of human B lymphoblastoid TK6 cells with 1,25-VD also led to a decrease in the level of constitutive expression of γH2AX and ATM-S1981^P^ (Figure 3). The overall effect of 1,25-VD in suppression of the level of constitutive phosphorylation of these proteins in TK6 cells was somewhat weaker than in A549 cells (Figures 1 and 2). However, as in the case of A549 cells, the S-phase cells were more affected by 1,25-VD than G_1_ or G_2_M phase cells in TK6 cultures and the suppressive effect of 1,25-VD on H2AX phosphorylation (13-18%) was more pronounced than on phosphorylation of ATM (8-12%).

**Figure 4:**
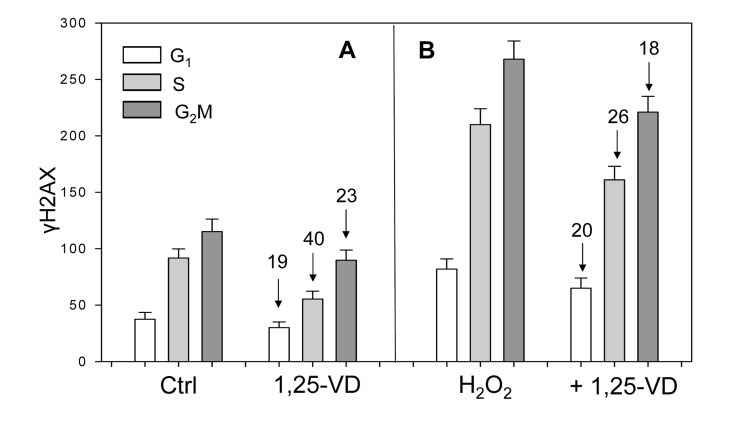
Effect of treatment of mitogenically stimulated proliferating human lymphocytes untreated or treated with H_2_O_2_ and 1,25-VD on the level of expression of γH2AX Human peripheral blood lymphocytes mitogenically stimulated with PHA for 48 h were untreated (Ctrl) or treated with 10 nM 1,25-VD for 24 h (A). Cells in another set of cultures (B) were either untreated with 1,25-VD but exposed to 200 μM of H_2_O_2_ for 1 h (H_2_O_2_) or were pretreated with 1,25-VD for 24 h and then treated with 200 μM H_2_O_2_ for 1 h (+H_2_O_2_). Expression of γH2AX was detected immunocytochemically and cell fluorescence was measured by flow cytometry. Through gating analysis (as shown in Figs. 1-3) the mean values (+SD) of fluorescence intensity for cells in G_1_, S and G_2_M phases of the cell cycle were obtained and plotted as bar graphs. Numbers above the arrows show the percent decline of the mean values of the 1,25-VD-treated cells compared to the cells not treated with 1,25-VD in the respective phases of the cycle.

We have also tested the effect of 1,25-VD on the level of constitutive expression of γH2AX and ATM-S1091^P^ in proliferating human lymphocytes (Figure 4). In the initial experiments we noticed that when 1,25-VD was added into cultures concurrent with PHA, lymphocyte stimulation, as expressed by the increases in cellular RNA content and progression through the cell cycle, was delayed compared to cells growing in the absence of 1,25-VD. This observation was consistent with prior studies that showed the suppressive effect of 1,25-VD on lymphocyte stimulation by a variety of mitogens [41-43]. However, when lymphocytes were already fully in the cell cycle 48 h following stimulation by PHA, the treatment with 1,25-VD did not alter their cell cycle progression over the subsequent 24 h. Under such conditions, we were able to test the effect of 1,25-VD on constitutive expression of γH2AX and ATM-S1981^P^ on cells which had fully entered the cell cycle similar to the cells stimulated by PHA growing in the absence of 1,25-VD (Figure 4A). Therefore, the results were not biased by the anti-proliferative effect of 1,25D which would have been apparent if 1,25-VD had been added earlier during lymphocytes stimulation by PHA.

Unlike the results on A549 and TK6 cells (Figures 1-3, scatterplots), the data on lymphocytes are presented as bar graphs (Figure 4). The effect of 1,25-VD on lymphocytes was similar to that its effect on A549 or TK6 cells. As it is evident, the level of expression of γH2AX was distinctly lower in the 1,25-VD treated cells (Fig 4A). The cells most affected were in S-phase, where γH2AX fluorescence was decreased by 40%, nearly twice as much as that of G_1_ or G_2_M cells. The level of expression of ATM-S1981^P^ was also decreased in 1,25-VD treated lymphocytes, but the decrease was less pronounced (10-18% across all phases of the cell cycle) compared to that of γH2AX (data not shown).

In another set of lymphocyte cultures, we explored whether 1,25-VD can affect expression of γH2AX upon induction of oxidative damage by H_2_O_2_. Towards this end, lymphocytes stimulated by PHA for 48 h and then treated with 1,25-VD for an additional 24 h were exposed to 200 μM H_2_O_2_ (+ 1,25-VD) for 60 min; parallel cultures of PHA stimulated lymphocytes were exposed to 200 μM H_2_O_2_ for 60 min without pretreatment with 1,25-VD (H_2_O_2_) (Figure 4B). The cells treated only with H_2_O_2_ had approximately a two-fold higher level of γH2AX expression compared with the untreated cells (Ctrl), consistent with the induction of oxidative DNA damage by the peroxide [40]. However, the effect of treatment with H_2_O_2_ in terms of a decline in expression of γH2AX was less pronounced in the cells exposed to 1,25-VD. Specifically, compared with the cells not treated with 1,25-VD, the treated cells exhibited an 18-26% lower level of γH2AX expression in which the S phase cells were more affected compared to G_1_ or G_2_M phase cells.

In addition to measuring the effect of 1,25-VD on expression of γH2AX and ATM-S1981^P^ in lymphocytes we have also measured the effect of this hormone on the intracellular level of ROS as detected by the lymphocytes' ability to oxidize dihydrofluorescein. This reagent (2,7-dichlorodihydrofluorescein diacetate; DCFH-DA) is permeant and non-fluorescent but when oxidized within the cell becomes fluorescent. Exposure of PHA-stimulated lymphocytes to 10 nM 1,25-VD for 24 h and 48 h (as described in Materials and Methods) reduced by about half (from 62 ± 3.5 to 32 ± 4.7 AU) or by nearly three-fold (from 62 ± 3.5 to 23 ± 4.7) their ability to oxidize DCFH-DA, respectively. However, we were not able to observe a significant reduction of DCFH-DA fluorescence in TK6 or A549 cells treated for 24 h with 2 nM or 10 nM 1,25-VD (data not shown).

## DISCUSSION

The present data show that the level of constitutive DNA damage signaling as measured by expression of γH2AX and ATM-S1981^P^ was decreased in A549 and TK6 tumor cells cultured in the presence of 1,25-VD for 24 - 48 h. Also decreased was the level of γH2AX and ATM-S1981^P^ in proliferating normal human lymphocytes cultured in the presence of 1,25-VD. These data suggest that both in normal proliferating lymphocytes as well as in A549 or TK6 tumor cells the level of constitutive DNA damage, likely induced by-products of oxidative metabolism was attenuated by 1,25-VD. Several mechanisms may be responsible for this effect.

It has been observed that the level of constitutive DNA damage signaling is more pronounced in S and G_2_M than in G_1_-phase cells [32-34]. This level was also seen to dramatically increase during mitogenic stimulation of lymphocytes [37]. Most likely, the high intensity of constitutive DNA damage signaling in S-phase cells is reporting DNA replication stress that occurs when replication forks encounter sites of DNA with oxidative damage. Replication stress, especially combined with mTOR activation can lead to elevated levels of DNA damage signaling (“pseudo-DNA damage response”) associated with cell senescence [39,44-47]. The suppressive effect on H2AX and ATM phosphorylation by 1,25-VD, as is evident in S-phase cells, suggests that in cells exposed to this hormone the intensity of replication stress is greatly diminished. Consistent with this is the evidence that in some cell types 1,25-VD suppresses Akt/mTOR signaling [48-50]. It should be noted, however, that in leukemic cells triggered by 1,25-VD to differentiation, the PI3K/Akt pathway is actually activated, making the association between these pathways and biological effects complex [51,52]. It is possible that activation of the PI3K/AKT/mTOR pathway takes place exclusively after induction of differentiation of promyelocytic leukemic (HL-60) cells whereas in other cell systems, when differentiation is not apparent, mTOR is inhibited by this hormone. Interestingly, inhibition of mTORC1 by the rapamycin analog RAD001 was shown to potentiate the effect of 1,25-VD to induce growth arrest and differentiation in AML cells, which prompted the authors to propose the strategy of concomitant administration of both 1,25-VD and an mTOR inhibitor in treatment of AML [53]. The anti-aging properties of vitamin D [54-58] would be consistent with its mTOR inhibitory activity [47,59].

The attenuation of DNA damage signaling by 1,25-VD in lymphocytes as seen in the present study would also be consistent with the anti-oxidant properties of this hormone [25-31]. Indeed we observed that exposure of lymphocytes to 1,25-VD led to a decrease in abundance of reactive oxidants that were detected by their ability to oxidize DCFH-DA. We were unable, however, to see a significant effect following exposure of TK6 or A549 cells for 24 h to 1,25-VD on oxidation of DCFH-DA. Yet, at the same time a reduction in the constitutive level of γH2AX in these cells was observed. While it is possible that the sensitivity of the DCFH-DA-ROS detection assay is inadequate to reveal minor changes in abundance of endogenous oxidants that still affect the level of constitutive H2AX phosphorylation, factors other than ROS induced by 1,25-VD may contribute to the lowering DNA damage signaling.

Our present findings are in accord with the recently published data of Ting et al., [60] in a mouse model system which show that 1,25-VD reduces the level of H2AX phosphorylation and ATM activation after induction of DNA damage by H_2_O_2_ or by N-nitroso-N-methylurea (NMU). Of interest are also their findings that 1,25-VD enhanced expression of DNA repair genes ATM and RAD50 and protected cells from genotoxic stress and malignant transformation. The mechanism advanced by the authors involves the cross-talk between ATM and 1,25-VD receptor (VDR). Specifically, activation of ATM, the early step in the DNA damage response pathway, in the presence of 1,25-VD leads to phosphorylation of VDR, which in turn induces transcriptional upregulation of ATM and RAD50 thereby promoting effective DSB repair [60]. With such interactions between ATM and VDR, one would expect that 1,25-VD may affect downstream pathways such as those leading to apoptosis and cell senescence, thereby providing a barrier to cancer development, and protecting genome integrity. The interactions between ATM and VDR may also be the mechanism that explains our data on reduction of constitutive H2AX phosphorylation (likely reporting the presence of DSBs) in A549 and TK6 cells in the absence of detectable effect of 1,25-VD on ROS. According to this mechanism [60] it is feasible that constitutive activation of ATM in the presence of 1,25-VD induces phosphorylation of VDR which further enhances expression of ATM and RAD50, leads to a more effective DNA repair, reduces frequency of DSBs and, as a consequence, dampens down the level of DNA damage signaling (γH2AX expression).

It has been reported that proliferation of cells from various solid tumor types including malignant melanoma [61], colon [62], breast [64] and prostate cancer [64] is inhibited by 1,25-VD. Vitamin D and its analogues are also widely recognized as potential anticancer drugs in the treatment of some hematological malignancies [2,9]. It is unclear if there is an association between molecular mechanisms responsible for the presently observed attenuation of constitutive DNA damage signaling and the previously reported effects of 1,25-VD on cell survival. For instance, in myeloid leukemia cells in culture 1,25-VD affects cell survival by upregulation of the AKT [52], and the KSR2 pathways [65]. Most recently, the upregulation of microRNA32 expression by 1,25-VD was shown to control the levels of the pro-apoptotic protein BIM [66]. These molecules could be components of a complex network that orchestrates cell survival at the level of DNA damage. However, the attenuation of DNA damage response (DDR) by 1,25-VD demonstrated here illustrates that DDR can be a barrier, or a pathway, to tumor progression, depending on the presence and nature of extracellular factors [67,68].

## MATERIALS AND METHODS

### Cells, Cell Treatment

Human lung carcinoma A549 cells, were obtained from the American Type Culture Collection (ATCC, Manassas,VA). Human B-cell lymphoblastoid TK6 cells were kindly provided by Dr Howard Liber of Colorado State University (Fort Collins CO). Human peripheral blood lymphocytes were obtained by venipuncture from healthy volunteers following informed consent according to Institutional guidelines, and isolated by density gradient centrifugation. A549 cells were cultured in Ham's F12K, TK6 and lymphocytes were cultured in RPMI 1640 with 2 mM L-glutamine adjusted to contain 1.5 g/L sodium bicarbonate supplemented with 10% fetal bovine serum (GIBCO/Invitrogen, Carlsbad, CA). Adherent A549 cells were grown in dual-chambered slides (Nunc Lab-Tek II), seeded with 10^5^ cells/ml suspended in 2 ml medium per chamber. TK6 cells and lymphocytes were grown in suspension; lymphocyte cultures were treated with the polyvalent mitogen phytohemaglutinin (Sigma/Aldrich; St Louis, MO) as described [37]. The active form of vitamin D, 1,25-VD, was kindly provided by Dr. Milan Uskokovic, and the stock solution was maintained in ethanol. During treatment with 1,25-VD the cells were in exponential phase of growth; control cells were treated with an equivalent concentration of ethanol. After exposure to 1,25-VD at various concentrations and for specified periods of time (as shown in figure legends) the cells were rinsed with phosphate buffered salt solution (PBS) and fixed in 1% methanol-free formaldehyde (Polysciences, Warrington, PA) for 15 min on ice The cells were then transferred to 70% ethanol and stored at -20 °C for up to 3 days until staining.

### Detection of H2AX Phosphorylation and ATM Activation

The cells were washed twice in PBS and with 0.1% Triton X-100 (Sigma) in PBS for 15 min and with a 1% (w/v) solution of bovine serum albumin (BSA; Sigma) in PBS for 30 min to suppress nonspecific antibody (Ab) binding. The cells were then incubated in 1% BSA containing a 1:300 dilution of phospho-specific (Ser139) γH2AX mAb (Biolegend, San Diego, CA) or with a 1:100 dilution of phospho-specific (Ser1981) ATM mAb (Millipore, Tamecula, CA). The secondary Ab was tagged with AlexaFluor 488 fluorochrome (Invitrogen/Molecular Probes, used at 1:200 dilution). Cellular DNA was counterstained with 2.8 μg/ml 4,6-diamidino-2-phenylindole (DAPI; Sigma). Each experiment was performed with an IgG control in which cells were labeled only with the secondary AlexaFluor 488 Ab, without primary Ab incubation to estimate the extent of nonspecific adherence of the secondary Ab to the cells. The fixation, rinsing and labeling of A549 cells was carried out on slides, and lymphocytes and TK6 cells in suspension. Other details have been previously described [39,40].

### Reactive Oxygen Species (ROS) Detection

Untreated cells as well as cells treated with vitamin D were incubated 60 min with 10 μM 2',7'-dihydro-dichlorofluorescein-diacetate (H2DCF-DA) (Invitrogen/ Molecular Probes) at 37°C. Cellular green fluorescence was then measured by flow cytometry. Following oxidation by ROS and peroxides within cells the non-fluorescent substrate H2DCF-DA is converted to the highly fluorescent derivative DCF [59].

### Analysis of Cellular Fluorescence

*A549 cells:* Cellular immunofluorescence representing the binding of the respective phospho-specific Abs as well as the blue emission of DAPI stained DNA was measured by Laser Scanning Cytometry (LSC) [70] (iCys; CompuCyte, Westwood, MA) utilizing standard filter settings; fluorescence was excited with 488-nm argon, helium-neon (633 nm) and violet (405 nm) lasers. The intensities of maximal pixel and integrated fluorescence were measured and recorded for each cell. At least 3,000 cells were measured per sample. Gating analysis was carried out as described in Figure legends. *TK6 cells and lymphocytes:* Cellular fluorescence was measured by using a MoFlo XDP (Beckman-Coulter, Brea, CA) high speed flow cytometer/sorter. DAPI fluorescence was excited with the UV laser (355-nm), AlexaFluor 488 and DCF with the argon ion (488-nm) laser. All experiments were repeated at least three times
